# “Setting the standard”: an analysis of different acquisition patterns for macular OCT-angiography

**DOI:** 10.1186/s40942-025-00653-w

**Published:** 2025-03-13

**Authors:** Ramkailash Gujar, Giulia Gregori, Rosa Dolz-Marco, Alessio Muzi, Jay Chhablani, Daniela Fruttini, Lorenzo Mangoni, Clara Rizzo, Cesare Mariotti, Marco Lupidi

**Affiliations:** 1 Cornea and Stem Cells Department, Dr. Shroff’s Charity Eye Hospital, Daryaganj, New Delhi India; 2 Department of Experimental and Clinical Medicine, Eye Clinic, Polytechnic University of Marche, Ancona, Italy; 3 Unit of Macula, Oftalvist Clinic, Valencia, Spain; 4 Eye Clinic, Humanitas-Gradenigo Hospital, Torino, Italy; 5 Department of Ophthalmology, UPMC Eye Center, University of Pittsburgh, Pittsburgh, USA; 6 Department of Medicine and Surgery, Section of Ophthalmology, University of Perugia, S. Maria della Misericordia Hospital, Perugia, Italy; 7 Ophthalmic Unit, Department of Neurosciences, Biomedicine, and Movement Sciences, University of Verona, Verona, Italy; 8 Fondazione Italiana Macula ETS, Di.N.O.G.Mi, University Eye Clinic, Genova, Italy

**Keywords:** Optical coherence tomography angiography, Scanning pattern, Lateral resolution

## Abstract

**Purpose:**

This study aimed to determine the optimal OCT angiography (OCT-A) scanning pattern using the SPECTRALIS HRA-OCT2 device to evaluate macular microvasculature perfusion in healthy subjects.

**Methods:**

Healthy subjects were imaged using the SPECTRALIS OCT-A Module (Heidelberg Engineering) with the following scanning protocols: 10ºX10º-512 ART 7 [P1], 10ºX10º-256 ART 5 [P2], 10ºX10º-512 ART 5 [P3], and 15ºX10º-256 ART 5 [P4], all centered on the macula. Vessel perfusion density (VPD) and vessel length density (VLD) of the superficial vascular complex (SVC) were calculated using ImageJ software to evaluate the differences between scanning patterns. Three additional 10ºx1º, ART 7 high-density images were also obtained using the in-built software (SP-X1701 Update 3, based on Heyex Software Version 1.9.215.0 H) in the macular area and the VPD and VLD for all the three10ºx1º pattern size images with the corresponding area of pattern 1 image[P1]. Two retinal specialists conducted a blind qualitative assessment of the foveal avascular zone and image quality.

**Results:**

Twenty eyes from 20 consecutive healthy patients were included in the study. The mean VPD for P1, P2, P3, and P4 were 35.60, 31.67, 31.18, and 31.16, respectively. Mean VLD for P1, P2, P3, and P4 were 7.54, 5.86, 6.74, and 4.40, respectively. Significant differences were found between P1 and the other patterns for both the VPD and VLD, but not between P2, P3, and P4. VPD and VLD for 10ºx1º high-density images were 33.20 and 4.61, respectively, with significant VLD differences compared to P1, but not for VPD. P1 scored the highest and P4 the lowest in the qualitative assessments.

**Conclusions:**

The 10ºX10º-512 ART 7 pattern showed statistically significant qualitative superiority and appeared optimal for blood flow detection with reduced noise in quantitative assessments.

## Introduction

Optical coherence tomography (OCT) angiography (OCT-A) provides visualization of retinal perfusion without the injection of contrast agents [1]. Dye-based angiographies are the gold standard for imaging retinal and choroidal vessels, and have been used for many years in clinical practice and clinical trials. However, they require the injection of a contrast agent and provide two-dimensional images, not being able to image individual layers of the vasculature at different depths. Conversely, OCT-A allows three-dimensional, high-resolution scans of the retinal vascular structure in a noninvasive manner. Owing to its depth-resolved nature, OCT-A allows the separate visualization of the superficial vascular complex (SVC), intermediate capillary plexus (ICP), deep capillary plexus (DCP), and choriocapillaris [1, 2]. OCT-A has some limitations, such as artifacts that can arise from OCT image acquisition, intrinsic characteristics of the eye, eye motion, image processing, and incorrect volume segmentation [3]. Previous studies have demonstrated good reproducibility and repeatability of quantitative measurements of the foveal avascular zone (FAZ) area, vessel perfusion density (VPD), and fractal dimension (FD) of the SVC and DCP in healthy eyes using different OCTA devices [4–9]. Nevertheless, it was shown that a comparison between OCT-A devices was nearly impossible because of the different OCT-A algorithms and scanning patterns [10]. Ishii et al. measured the FAZ area on *en-face* OCT-A images using a manual approach and an automated software-assisted method, showing that both methods were comparable [11]. Hosari S et al. showed a good intra-visit reliability values of the macular microvascular measurements including VPD and FAZ on SPECTRALIS HRA-OCT2 (Heidelberg Engineering, Heidelberg, Germany) OCT-A images in combination with a custom built software [12]. It has been hypothesized that the detection of blood flow on OCT-A relies on two factors: the distance between two consecutive B-scans, and the number of averaged frames per B-scan. Freund et al. (2018) and Lupidi et al. (2019) showed that high-density volume scans provide more blood flow information than standard volumes and correlate it with retinal microstructure visualization [13, 14]. Therefore, it is essential to understand the reliability of different scanning protocols for OCT-A and their sensitivity to retinal microvascular measurements. Choosing the correct OCT-A acquisition protocol is essential for several reasons. It improves diagnostic accuracy; an inappropriate protocol can result in suboptimal image quality, limiting the identification of subtle physiological variations or early anomalies. Data comparability: Using a standardized protocol ensures that data are comparable over time or with those of other patients. Prevention of artifacts: This avoids the risk of artifacts leading to an abnormal interpretation of the images. Optimization of time and resources. It also enables the representation of exact human anatomy, which can be used as a basis for developing statistical models or artificial intelligence algorithms [15, 16]. To our knowledge, this is the first study to evaluate different OCT-A scanning protocols for quantitative and qualitative assessments using a single OCT-A device in healthy subjects. The purpose of this study was to assess the optimal scanning pattern on the SPECTRALIS HRA-OCT2 device by comparing different approaches with variable lateral resolutions.

## Methods

### Study design

This observational, cross-sectional study involving healthy eyes was conducted at the Eye Clinic of the Azienda Ospedaliero-Universitaria delle Marche (Ancona, Italy). Prior approval was obtained from the Marche Institute Review Board (IRBs), and the procedures conformed to the tenets of the Declaration of Helsinki. Written informed consent was obtained from all participants prior to sequential OCT imaging.

### Study population

Consecutive healthy patients who underwent a comprehensive ophthalmology assessment were included. Best-corrected visual acuity, slit-lamp biomicroscopy, intraocular pressure measurement, fundus evaluation, and OCTA were performed between November 2023 and January 2024. Inclusion criteria for this study included eyes with 20/20 visual acuity or better with no history or clinical evidence of any retinal disease or systemic illness, and all subjects older than 18 years of age. Exclusion criteria included any history or clinical evidence of retinal disease, glaucoma, previous ocular surgery or laser, and refractive error of +/-3 diopters or more. We also excluded eyes with limited image quality on OCTA (quality index [QI] < 30 dB) due to poor fixation or media opacities.

### OCTA image acquisition

OCTA images were obtained using SPECTRALIS HRA + OCT2 (Heidelberg Engineering, Heidelberg, Germany), which was able to acquire 85,000 A-scans per second with 3.9-µm axial and 6-µm lateral resolutions. This device uses a probabilistic amplitude decorrelation algorithm and a light source of 870 nm, with a bandwidth of 50 nm. For additional details regarding the device or the OCTA algorithm, we refer interested readers to our previously published studies [17, 18]. TruTrack^®^ active eye-tracking technology was used to reduce motion artifacts. A high-resolution structural volume scan centered on the fovea was captured (the automated real-time [ART] mode was set at 5 or 7 frames/scan).

**Fig. 1:**
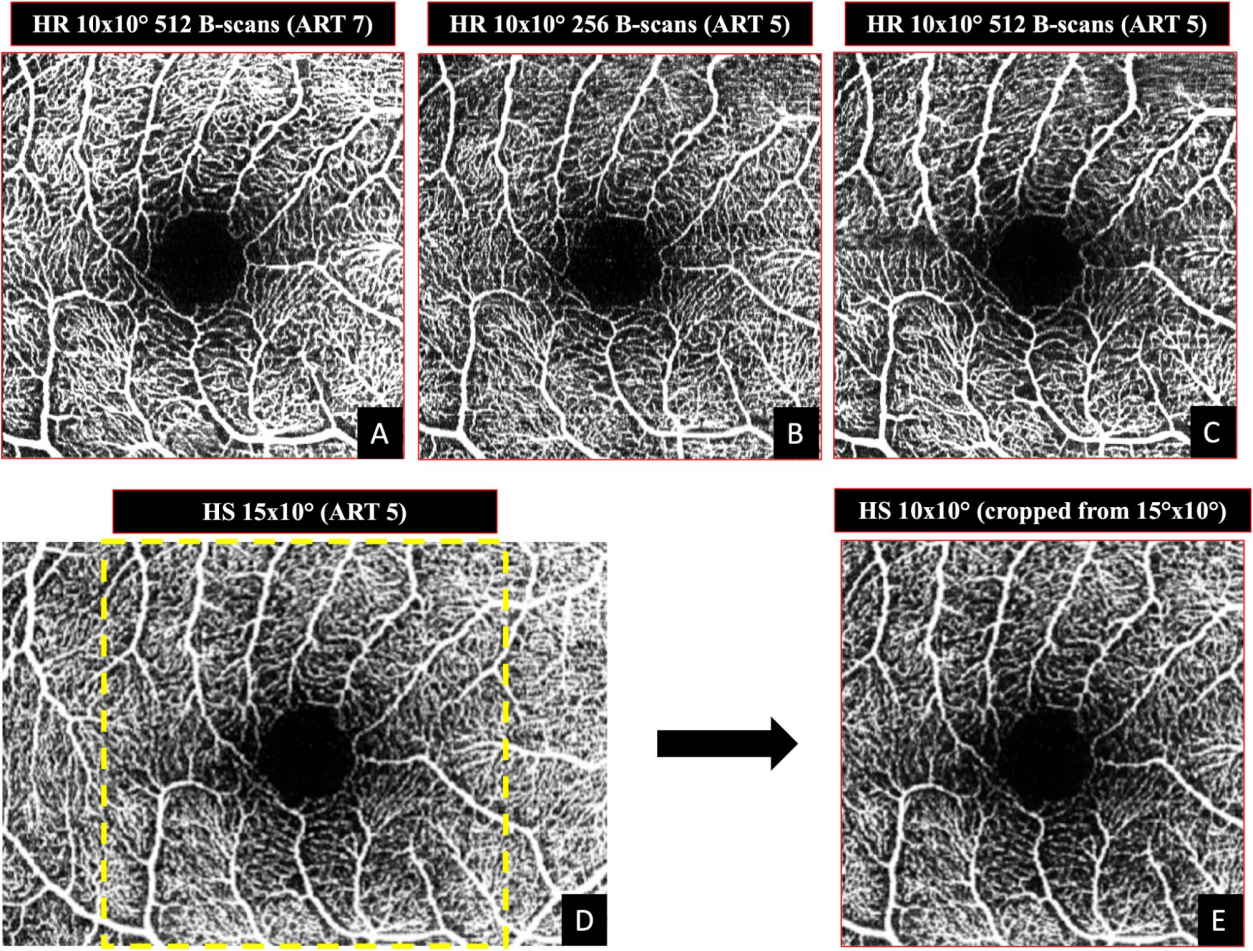
Example of Pattern 1[P1]: 10ºX10º (2.9 × 2.9 mm); 512 a-scans x 512 b-scans ART 7 (10ºX10º- 512 ART 7) figure **A**; Pattern 2 [P2]: 10ºx10º, 256 a-scans x 256 b-scans ART 5 (10ºX10º- 256 ART 5) Figure **B**; Pattern 3 [P3]: 10ºx10º pattern, 512 a-scans x 512 b-scans ART 5 (10ºX10º- 512 ART 5) Figure **C**. Pattern 4 [P4]: 15ºx10º (4.4 × 2.9 mm); 768 a-scans x 256 b-scans; ART 5 (15ºX10º-256 ART 5) Figure **D**. Figure D cropped to a 10°×10° field of view for direct comparison with the other images.” Figure **E**

The OCTA patterns obtained in each eye included: Pattern 1[P1]: 10ºX10º (2.9 × 2.9 mm); 512 a-scans x 512 b-scans ART 7 (10ºX10º- 512 ART 7); Pattern 2 [P2]: 10ºx10º, 256 a-scans x 256 b-scans ART 5 (10ºX10º- 256 ART 5); Pattern 3 [P3]: 10ºx10º pattern, 512 a-scans x 512 b-scans ART 5 (10ºX10º- 512 ART 5). Pattern 4 [P4]: 15ºx10º (4.4 × 2.9 mm); 768 a-scans x 256 b-scans; ART 5 (15ºX10º-256 ART 5). Three additional 10ºx1º images, ART 7, were obtained using the in-built software (SP-X1701 Update 3, based on Heyex Software Version 1.9.215.0 H, Heidelberg Engineering, Heidelberg, Germany) in the macular area that allows multiple scans times in 1º, and after which high-quality high-density scans were obtained [17]. Each 10ºx1º image included 300 B-scans in 1º area (10ºx1º-300-7 ART). To summarize, the acquisition patterns P1, P2, P3, and three additional 10ºx1º images were obtained using OCT-A at high resolution (HR). In contrast, the P4 pattern was acquired at high speed (HS). This specific pattern had a larger field of view (15ºx10º) to maintain the same number of pixels as the HR images. To enable an optimal comparison, the P4 images were cropped to match the dimensions of the other images, allowing for proper overlay and alignment. (Fig. 1) OCTA images from the four different scanning patterns were reviewed by a retina specialist (ML) to detect potential segmentation errors and evaluate the quality of the scans. The OCTA *en face* reconstruction of the SVC was obtained for each of the four different patterns. We used the automated segmentation provided by the built-in software viewer (Heyex Software version 1.9.201.0; Heidelberg Engineering) with manual correction when needed. The SVC boundaries were set at the internal limiting membrane and inner margin of the inner plexiform layer. The built-in projection artifact removal tool was used to avoid potential artifacts, and the fixed contrast was set at 1.5. The OCTA *en face* reconstruction of the DCP and choriocapillaris were not evaluated in the present study; thus, the influence of projection artifacts was avoided. The alignment of the images was assessed by evaluating the overlap of the retinal blood vessels to ensure proper registration of the corresponding vascular structures. Visual inspection was performed to verify the accuracy of the alignment.

### Quantitative and qualitative assessment of OCTA images

All OCTA *en face* reconstructions of the SVC for each scanning pattern (four images per eye) were processed for qualitative and quantitative assessments.

**Fig. 2:**
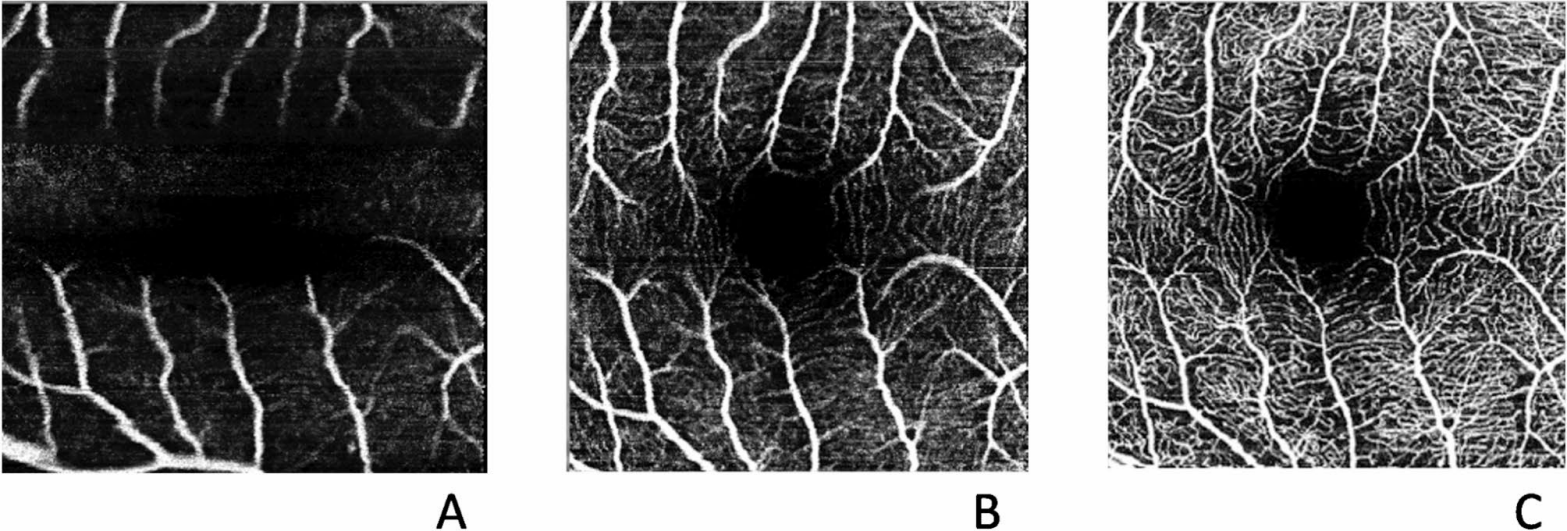
Example of Grade images of poor quality **A**, average quality **B**, and good quality **C**

Qualitative assessment was performed independently by 2 retinal specialists (ML and RDM). To perform a blind reading, the images were presented randomly without the pattern label. The continuity of the FAZ was assessed and graded as < 90º (1), 90º-180º (2), 180º-270º (3), 270º-360º (4). The image quality was also graded as poor (1), average (2), and good (3) for each one of the different scan pattern. (Fig. 2)

**Fig. 3:**
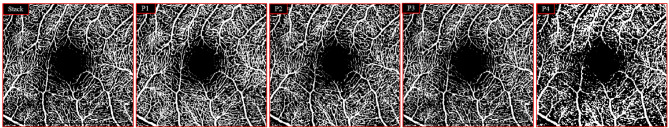
The stack image and the different scanning patterns used are shown: Pattern 1[P1]: 10ºX10º(2.9 × 2.9 mm); 512 a-scans x 512 b-scans; ART 7(10ºX10º- 512 ART 7), Pattern 2 [P2]: 10ºx10º; 256 a-scans x 256 b-scans; ART 5(10ºX10º- 256 ART 5), Pattern 3 [P3]: 10ºx10º pattern; 512 a-scans x 512 b-scans; ART 5 (10ºX10º- 512 ART 5). Pattern 4 [P4]: 15ºx10º (4.4 × 2.9 mm); 768 a-scans x 256 b-scans; ART 5(15ºX10º-256 ART 5)

For quantitative assessment, VPD and vessel length density (VLD) were computed using ImageJ software version 1.48 (National Institutes of Health, Bethesda, Maryland, USA) [19]. Prior to the calculation of VPD and VLD, four different images of each eye were stacked, aligned using a plugin *stack-reg* in ImageJ software, and cropped to ensure that the same area was evaluated. Binarization of the stack image was performed by selecting the default method and background light in ImageJ (Fig. 3). After the stack process, the images were separated again to calculate the VPD and VLD using the specific vessel analysis plugins. Similarly, we quantified the VPD and VLD for all three 10ºx1º pattern size high-density images with the corresponding area of the pattern 1 image [P1]. In particular, the VPD was defined as the ratio of the area occupied by vessels divided by the total area. VLD was calculated by converting all vessels into a single pixel-wide structure(skeletonization), thereby removing the effect of larger vessels on the overall vessel density. VLD was measured in mm⁻¹.

### Statistical analysis

All variables were described by their mean and standard deviation (SD). Statistical significance was set at *p* value < 0.05. All statistical analyses were performed using SPSS statistical software version 21(SPSS Inc., IBM Company, Chicago, IL). Friedman test was used to compare mean values. Bland-Altman analysis was performed by setting the limits of agreement to two standard deviations.

## Results

### Demographic data

Twenty eyes from 20 consecutive healthy patients (12 males) were included in the present study. The mean age was 29.0 ± 6.5 years.

### Qualitative data

In the qualitative assessment, the inter-grader comparison showed no statistically significant differences in the evaluation of the images by the two operators for the continuity of the FAZ in the various patterns (*p* = 0.9290). Statistically significant differences were reported in image quality grading for P2 (*p* = 0.0098) and P4 (*p* = 0.0022). Grade 1(RDM) was compared to grade 2 (ML).

In the intra-grader comparison, statistically significant differences were reported for the FAZ (grade 1, *p* = 0.00010; grade 2, *p* = 0.00348) and image quality (grade 1 *p* = 0.0001, grade 2, *p* < 0.0001). We also found that image P1 had the highest score and image P4 had the lowest score from both graders in both the FAZ and image quality assessments.

### Quantitative data

**Fig. 4:**
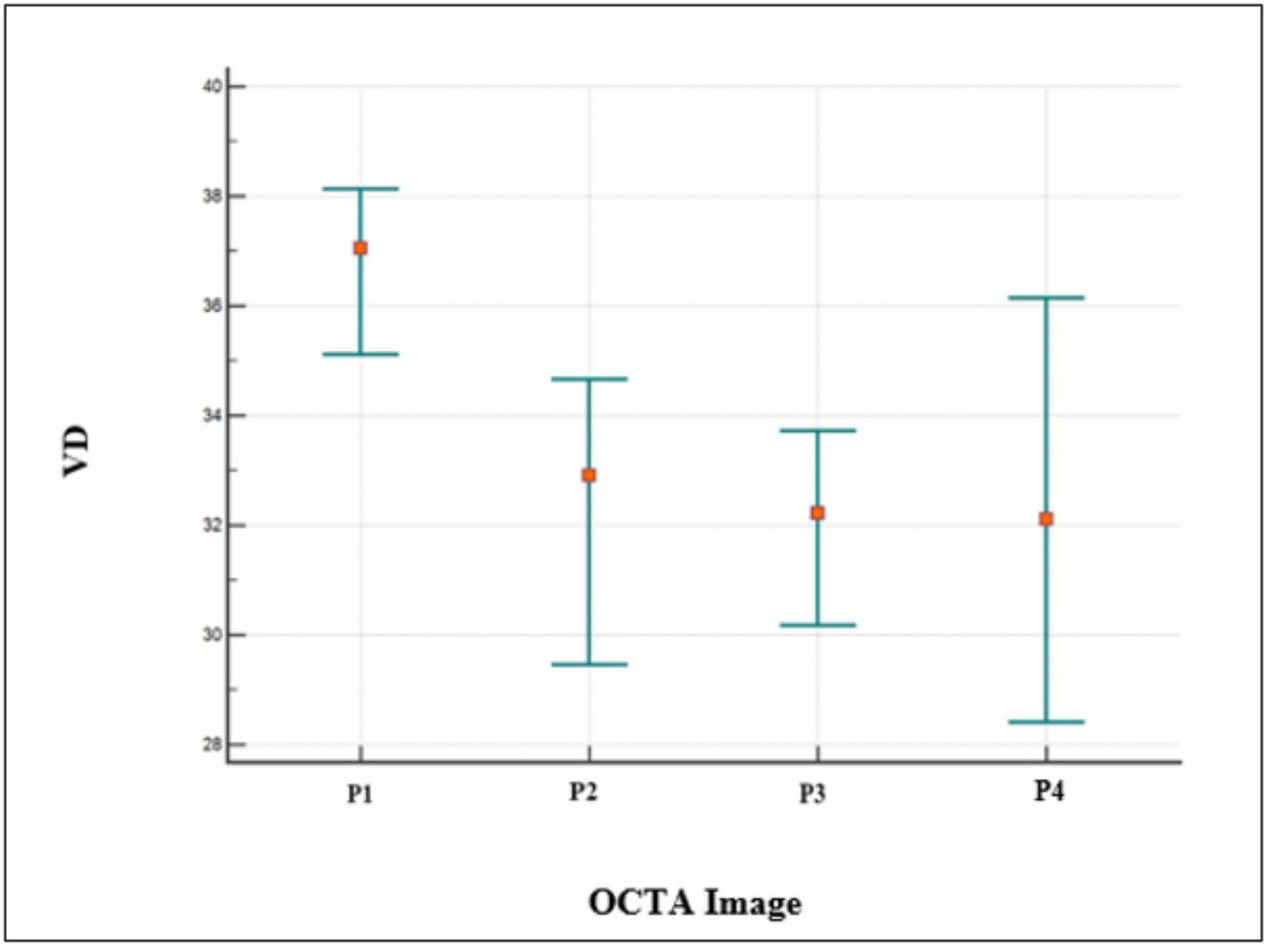
The plot shows statistically significant differences between the P1 scan pattern and other scanning patterns (P2, P3, and P4), but no statistically significant differences between P2, P3, and P4 for vessel perfusion density (VPD)

**Fig. 5:**
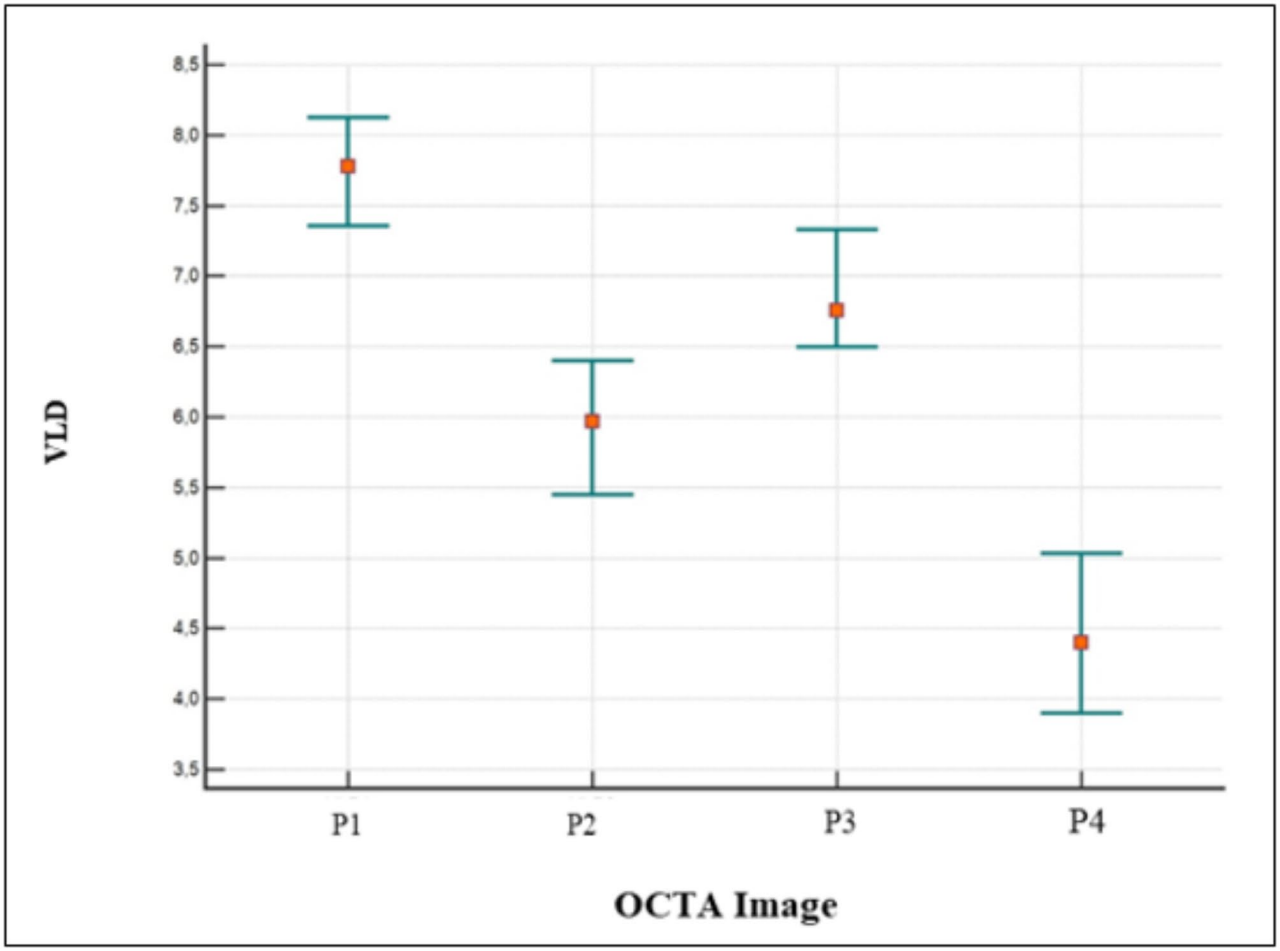
The plot shows statistically significant differences between the P1 scan pattern and other scanning patterns, P2, P3, and P4, but no statistically significant differences between P2, P3, and P4 for vessel length density (VLD)

In the quantitative assessment, the mean VPD values of the *en-face* images P1, P2, P3, and P4 were 35.60, 31.67, 31.18, and 31.16, respectively. The mean VLD values for P1, P2, P3, and P4 were 7.54, 5.86, 6.74, and 4.40, respectively. A statistically significant difference was reported between P1 and P2, P3, and P4 for both VPD (p˂0.0001) and VLD (p˂0.0001)). No statistically significant differences were found between P2, P3, and P4 values for the VPD (p˃0.05)) but there was a significant difference between P1 and P2 (p˂0.0001), P1 and P3 (*p* = 0.0042), P1 and P4 (p˂0.0001), P2 and P3 (p˂0.0011), P2 and P4(p˂0.0001) and P3 and P4(p˂0.0001) for the VLD (Figs. 4 and 5).

**Fig. 6:**
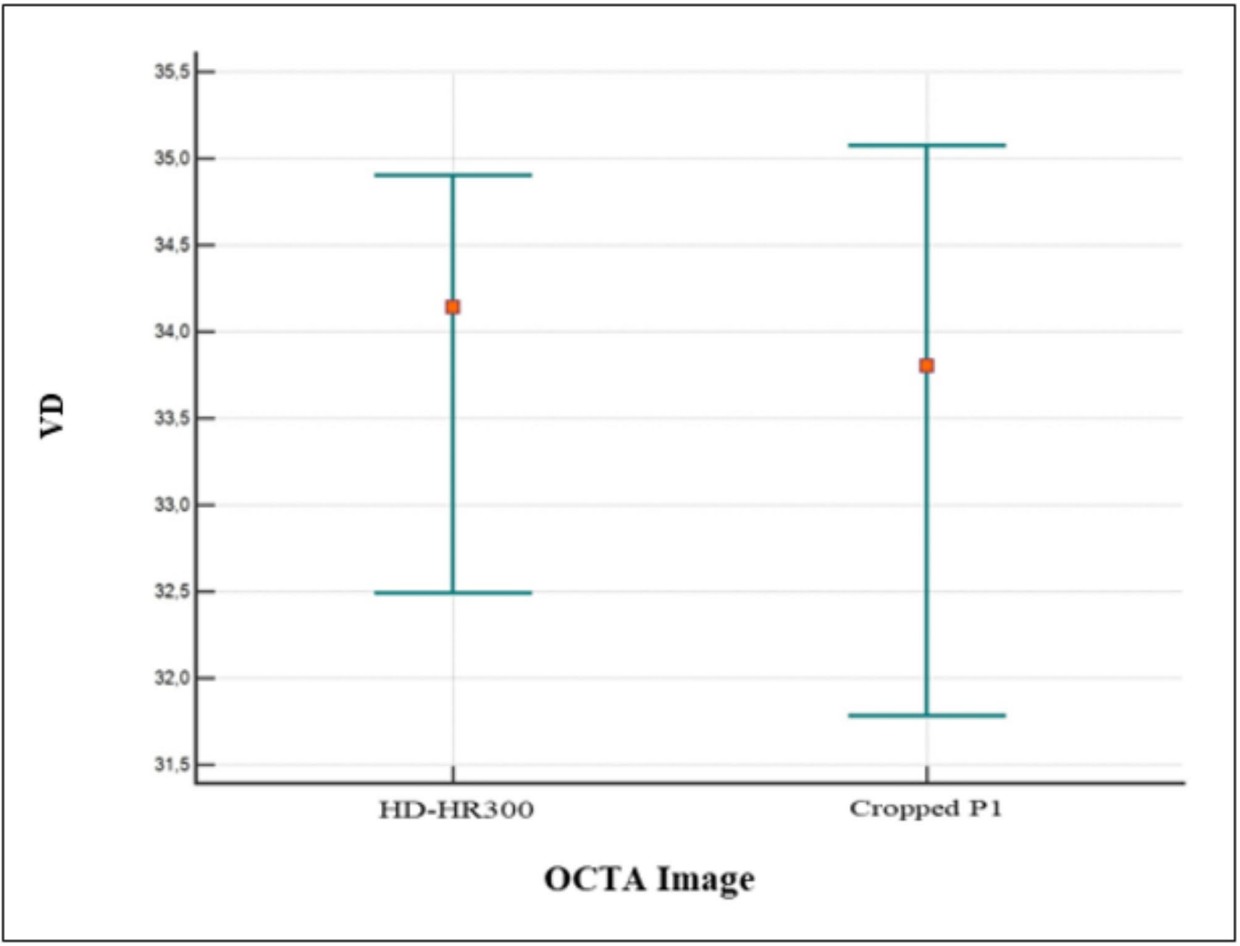
The plot shows no statistically significant difference between the 10ºx1º high-density image and the corresponding cropped area from the P1 image for VPD (*p* = 0.72)

**Fig. 7:**
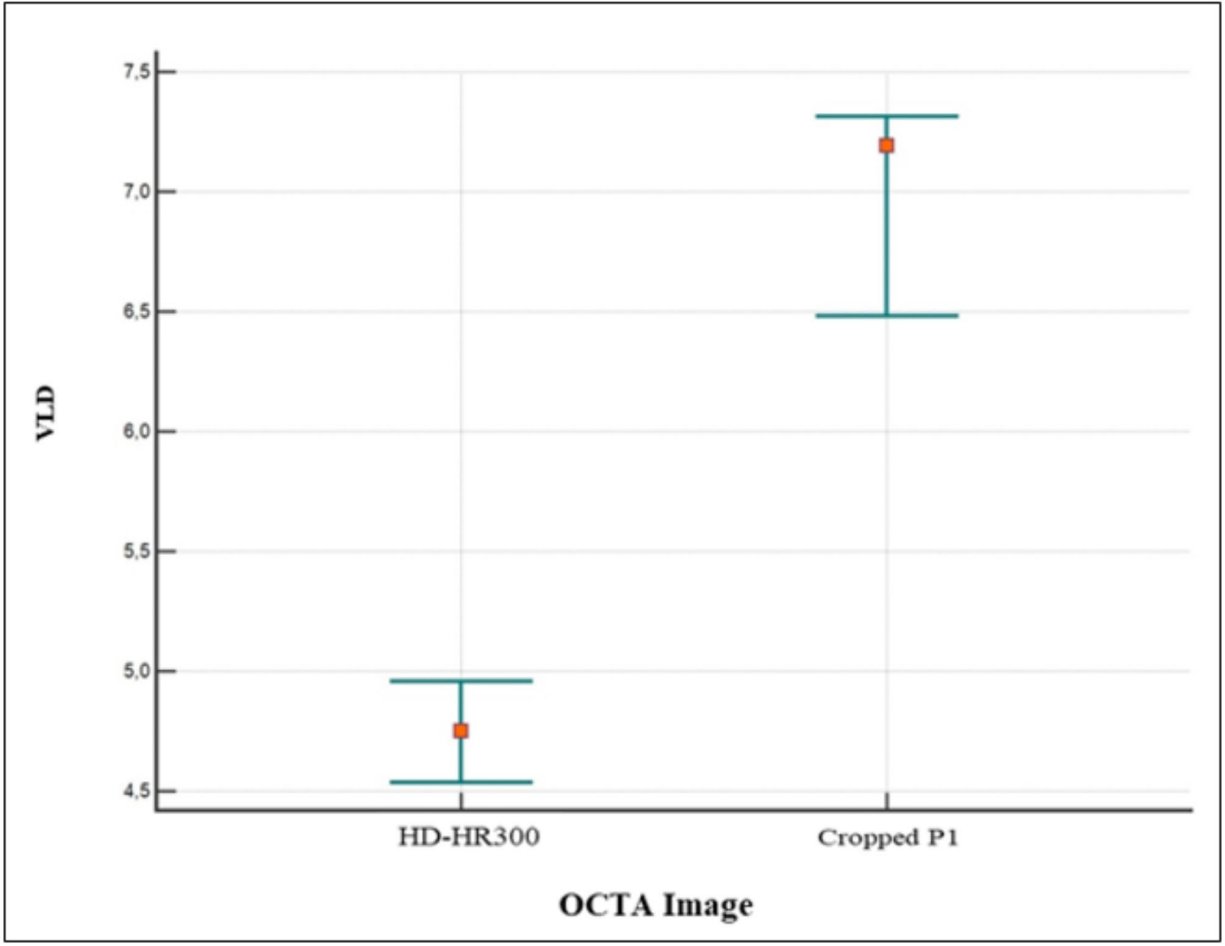
The plot shows a statistically significant difference between the 10ºx1º high-density image and the corresponding cropped P1 image for VLD (*p* < 0.0001)

In addition, the mean VPD values of 10ºx1º high density image(10ºx1º-HR-300-7ART) and cropped image P1(corresponding to a 10ºx1º area) were 33.20 and 32.91°, respectively. The mean VLD values of the 10ºx1º high-density image (10ºx1º-HR-300-7ART) and cropped image P1(corresponding a to 10ºx1º area) were 4.61 and 6.885. A statistically significant difference was reported between both 10ºx1º high-density image and cropped P1 images for VLD (*p* < 0.0001), but no statistically significant difference was observed for VPD (*p* = 0.72) (Figs. 6 and 7).

## Discussion

Since the development of OCTA, the focus has been on optimizing *en-face* visualization using different computer-generated algorithms. All OCTA devices enable en face reconstruction using a variety of scanning patterns. However, there is currently no consensus on the optimal pattern for capturing the detailed vascular flow characteristics. Therefore, in the present study, we compared different OCTA scanning patterns using a single SPECTRALIS HRA-OCT2 device for each healthy eye and assessed quantitative and qualitative information. In addition, correlations between different scanning patterns and high-density images were assessed. We could not find any differences in the FAZ evaluation during the inter-grader comparison in the various pattern images; however, differences in image quality were reported. Similarly, a difference was noticed in the intra-grader comparison for both FAZ and image quality. In this qualitative assessment, scanning pattern image P1 (10ºX10º- 512 ART 7) had the highest score for both the FAZ and image quality assessments from both graders. In the quantitative assessment, we observed that image P1 showed higher values than images P2, P3, and P4, whereas no differences were observed among P2, P3, and P4 images for both the VPD and VLD. We also did not find any differences in the comparison between P1 (highest-value image) and high-density images.

Our results showed that in the quantitative assessment, image P1 was shown to be an optimal scanning approach for detecting blood flow compared to the other analyzed scanning patterns in the same device and seemed to suffer less from noise. Uji et al. [20] demonstrated that *en face* averaging can significantly improve the *en face* OCTA image quality and impact quantitative measurements. Similarly, Sakamoto et al. [21] found that averaging more than four SD scans resulted in a significant improvement in the quality of images and visualization to distinguish retinal structures. The P1 images had a higher lateral resolution than P2, P3, and P4, isotropic lateral resolution, and a higher number of repeats per B-scan (ART7), showing that high lateral resolution with a high ART plays an essential role in the acquisition of high-quality OCTA images with a possible impact on clinical information. However, the acquisition of high-quality images may have the limitation of a longer acquisition time. The decision on the need to acquire faster OCTA scans versus high-quality OCTA images should be made during the planning of clinical studies or clinical trials, including OCTA volume scans. However, we found no differences between the high-density images created with lateral averaging and the P1 images. The clinical value of these high-density images has been previously demonstrated [13, 14]; however, our results suggest that lateral averaging on dense OCTA volume scans may play a role in increasing the B-scan image quality with a lower effect on *en face* reconstruction. In addition, P1 had an optimal lateral resolution (6 μm) to show detailed *en face* OCTA images.

Although the study was conducted in healthy subjects, the benefits of a proper OCT-A image acquisition protocol also have clinical implications. Determining the presence or absence of maculopathies is a challenging task. For example, in diabetic retinopathy, an inadequate protocol might fail to capture early microaneurysms or capillary dropout in the deep capillary plexus, leading to delayed diagnosis and intervention [22]. Similarly, in age-related macular degeneration (AMD), a poor scan resolution could obscure the detection of choroidal neovascularization, affecting treatment decisions [23]. Using a standardized protocol ensures consistent imaging data for reliable comparisons over time. For example, OCTA can be used to monitor treatment response to MNV, but without standardization, variations in scan density, field of view, or segmentation parameters could compromise the consistency of quantitative biomarkers [1]. Optimizing the scan speed, reducing patient movement, and ensuring accurate retinal layer segmentation improve the image quality. For example, in highly myopic patients, incorrect segmentation may misrepresent vascular structures, leading to erroneous interpretations. To our knowledge, this study is the first to quantitatively and qualitatively evaluate the VPD and VLD of *en-face* OCT angiograms of healthy subjects using different scanning patterns on a single OCTA device. The selection of an optimal scan pattern is key and depends on the information that needs to be gathered. However, this study also has some limitations, including a small sample size and the lack of timing evaluation to assess the impact of acquiring high-quality images on the clinical workflow. In addition, only *en face* OCTA information was assessed in the present study, and future studies evaluating B-scan information are warranted to fully assess the impact of scan selection.

## Conclusion

Our qualitative assessment showed a statistically significant superiority for P1 (10ºX10º- 512 ART 7) compared with the other scanning patterns, highlighting the importance of high lateral resolution with a high number of B-scan repeats. In the quantitative assessment, P1 was shown to be an optimal scanning approach for detecting blood flow and seemed less affected by noise. Careful selection of the scan pattern is needed when evaluating the OCTA data, especially in protocolized clinical studies or clinical trials.

## Data Availability

No datasets were generated or analysed during the current study.

## Abbreviations

OCT: Optical coherence tomography
OCT: Optical coherence tomography angiography
SVC: Superficial vascular complex
ICP: Intermediate capillary plexus
DCP: Deep capillary plexus
FAZ: Foveal vascular zone
VPD: Vessel perfusion density
FD: Fractal dimension
VLD: Vessel length density
SD: Standard deviation
